# Hippocampal ferroptosis is involved in learning and memory impairment in rats induced by microwave and electromagnetic pulse combined exposure

**DOI:** 10.1007/s11356-023-28280-8

**Published:** 2023-06-22

**Authors:** Yunfei Lai, Haoyu Wang, Xinping Xu, Ji Dong, Yiwei Song, Haixia Zhao, You Wu, Li Zhao, Hui Wang, Jing Zhang, Binwei Yao, Yong Zou, Hongmei Zhou, Ruiyun Peng

**Affiliations:** Beijing Institute of Radiation Medicine, Beijing, 100850 China

**Keywords:** Microwave, Electromagnetic pulse, Combined exposure, Learning and memory impairment, Ferroptosis, Hippocampus

## Abstract

Microwave (MW) and electromagnetic pulse (EMP) are considered environmental pollutants, both of which can induce learning and memory impairments. However, the bioeffects of combined exposure to MW and EMP have never been explored. This paper aimed to investigate the effects of combined exposure to MW and EMP on the learning and memory of rats as well as its association with ferroptosis in the hippocampus. In this study, rats were exposed to EMP, MW, or EMP and MW combined radiation. After exposure, impairment of learning and memory, alterations in brain electrophysiological activity, and damage to hippocampal neurons were observed in rats. Moreover, we also found alterations in ferroptosis hallmarks, including increased levels of iron, lipid peroxidation, and prostaglandin-endoperoxide synthase 2 (PTGS2) mRNA, as well as downregulation of glutathione peroxidase 4 (GPX4) protein in the rat hippocampus after exposure. Our results suggested that either single or combined exposure to MW and EMP radiation could impair learning and memory and damage hippocampal neurons in rats. Moreover, the adverse effects caused by the combined exposure were more severe than the single exposures, which might be due to cumulative effects rather than synergistic effects. Furthermore, ferroptosis in the hippocampus might be a common underlying mechanism of learning and memory impairment induced by both single and combined MW and EMP exposure.

## Introduction

Electromagnetic radiation is well known as one of the most common environmental pollution sources in modern society (World Health Organization 2022). Given that it is ubiquitous in everyday life with the increasing use of wireless equipment, the potential health hazards induced by electromagnetic radiation have aroused public concern (Lai et al. 2021). Microwave (MW) and electromagnetic pulse (EMP) are two kinds of electromagnetic waves with specific physical properties. MW is defined as electromagnetic waves with frequencies ranging from 300 MHz to 300 GHz (Lai et al. 2021). EMP is a high voltage pulse with a broad bandwidth, high energy, and a short pulse duration (Tian et al. 2020). Previous studies suggested that either MW or EMP single exposure could induce learning and memory impairment (Tian et al. 2020; Li et al. 2019; Wang et al. 2017; Hao et al. 2018; Deshmukh et al. 2015; Li et al. 2022; Mumtaz et al. 2022; Wang et al. 2023a, b; Hao et al. 2022; Lai et al. 2022). However, real public and occupational environments are usually filled with various types of electromagnetic waves. Therefore, several preliminary studies have been carried out to investigate the effects of combined electromagnetic exposure on learning and memory (Tan et al. 2017; Shirai et al. 2017; Zhu et al. 2021). Most of these previous studies focused on the combination of MW radiation with different frequencies. However, the learning and memory effects induced by MW and EMP combined exposure have never been investigated.

The hippocampus is one of the brain regions sensitive to electromagnetic radiation (Zhi et al. 2017). The status of hippocampal neurons is known to be closely associated with learning and memory function (Bettio et al. 2017; Eichenbaum 2000). Previous studies have reported that learning and memory impairment and hippocampal structural damage induced by combined exposure to electromagnetic radiation might rely on the activation of oxidative stress or energy metabolism (Tan et al. 2017; Zhu et al. 2021). However, the mechanisms of learning and memory impairment and hippocampal damage induced by combined exposure are still unclear. Ferroptosis, a recently proposed novel type of regulated cell death (RCD) that is characterized by iron-dependent lipid peroxidation, provides us with a novel way to understand the underlying mechanism. Compared with other forms of RCD, ferroptosis possesses unique morphological, biochemical, and genetic features (Dixon et al. 2012; Tang et al. 2021). Swollen or condensed mitochondria with reduced cristae are considered to be the morphological changes of ferroptosis (Tang et al. 2021). Moreover, the accumulation of iron and lipid peroxides are the most important biochemical features in ferroptosis (Dixon et al. 2012; Tang et al. 2021). Furthermore, prostaglandin-endoperoxide synthase 2 (PTGS2) mRNA upregulation is considered to be an important genetic characteristic in ferroptosis (Tang et al. 2021). Additionally, glutathione peroxidase 4 (GPX4) is a classic and core regulator in ferroptosis, and reduces lipid hydroperoxides to nontoxic lipid alcohols (Stockwell 2022; Yang et al. 2014; Ingold et al. 2018).

The purpose of this work was to investigate the effects of MW and EMP combined exposure on learning and memory functions as well as their association with ferroptosis in the hippocampus. To achieve these goals, studies based on rat models were conducted. In this study, the rats were exposed to EMP, MW, or EMP and MW combined radiation. Then, the effects of combined exposure on learning and memory abilities and brain electrical activities in rats were evaluated by the Morris water maze (MWM) test and electroencephalogram (EEG), respectively. Moreover, pathological changes in hippocampal microstructure and ultrastructure were observed after exposure. Furthermore, the association of ferroptosis in the hippocampus and the impaired learning and memory ability induced by combined exposure was also investigated.

## Materials and methods

### Animals

All studies involved in this work were approved by the Ethics Committee of Beijing Institute of Radiation Medicine (no. IACUC-DWZX-2020–780). All procedures were conducted according to the National Institute of Health Guide for the Care and Use of Laboratory Animals. Male Wistar rats (200 ± 20 g) were obtained from the Beijing Vital River Laboratory Animal Technology Co., Ltd. (Beijing, China) and kept at a constant temperature (24 ± 2 °C) and humidity (60%) under a 12 h light/dark cycle. All animals had access to food and water ad libitum. Rats were randomly divided into four groups (n = 197): Sham exposure group (Sham, n = 46), EMP exposure group (EMP, n = 50), MW exposure group (MW, n = 51), and EMP and MW combined exposure group (EMP + MW, n = 50).

### MW and EMP exposure system

**Fig. 1:**
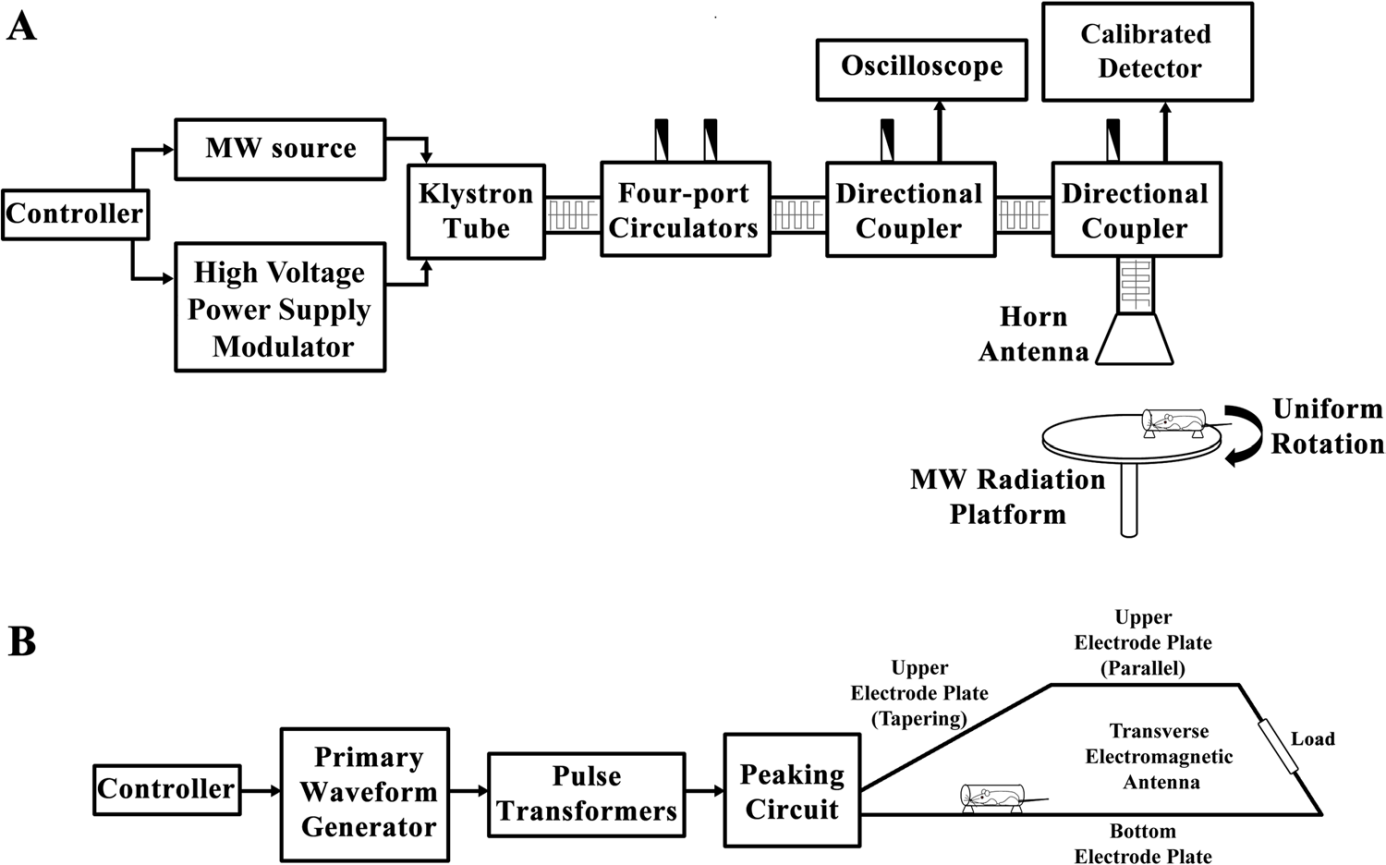
Schematic diagram of MW and EMP radiation systems and exposure scenarios. (**A**) Schematic diagram of the MW radiation setup. (**B**) Schematic diagram of the EMP radiation setup

An MW apparatus with a centre frequency of 1.5 GHz described in previous literature (Zhu et al. 2021) were implemented in this study to generate microwave radiation. The detailed composition of the MW apparatus is shown in Fig. 1 A. A vertical polarization bounded wave EMP simulator developed by the Beijing Institute of Radiation Medicine was used in this study to generate EMP radiation. The detailed composition of the EMP simulator is shown in Fig. 1 B. Both the MW and EMP exposure apparatus were placed in electromagnetic shield chambers. The inner walls of the chambers were covered with pyramid-shaped microwave absorbers to minimize reflections (> 45 dB).

For the single exposure group, rats were whole-body exposed to either MW radiation (average power densities = 30 mW/cm^2^, repetition frequency = 200 pps, pulse width = 500 ns, exposure time = 15 min) or EMP radiation (peak intensity = 400 ± 25 kV/m, repetition frequency = 1 Hz, number of pulses = 400). In the combined exposure group, rats were whole-body exposed to EMP radiation and then immediately exposed to MW radiation with the same parameters as the single exposure. The interval time between EMP and MW exposures was short and negligible. As shown in Fig. 1 A, during MW exposure, rats were awake and held in specifically designed fixation boxes made of plexiglass and free of metal. The fixation boxes were placed on the MW radiation platform, which was uniformly rotated during the exposure. As shown in Fig. 1 B, during EMP exposure, rats were also held in the fixation boxes and placed at the tapering section of the transverse electromagnetic antenna. Rats in the Sham group were treated the same as the EMP + MW group except for exposure.

The SAR value of MW exposure and the peak electromagnetic field strength of EMP exposure in rats were calculated using the finite difference time domain (FDTD) method in the simulation environment of Sim4life V7.0 (Zurich Med. Tech., Switzerland) based on a rat digital model. The permittivity and conductivity of different tissues were based on the IT’IS 4.0 database. All simulations were conducted on a workstation with the following configurations: CPU: Xeon E3-1225 3.2 GHz (Intel, USA), RAM: 16 GB, GPU: QUADRO K2200 (Nvidia, USA). The SAR value in rats was 10.57 W/kg, and the peak electromagnetic field strength in rats was 11.65 kV/m.

### Rectum temperature measurement

The rectum temperatures of the rats were measured before and immediately after MW exposure, EMP exposure, and combined exposure using a fibre optic thermometer (FOT-m, FISO, Canada).

### Morris water maze (MWM)

The MWM test was used to examine learning and memory in rats. The MWM test was performed according to previous literature (Vorhees and Williams 2006). Briefly, a black circular pool (1.5 m in diameter) filled with water and computer tracking software (Anymaze 6.1, Stoelting, USA) were used in the test. Rats were trained for three consecutive days before exposure. At 1 d, 2 d, 3 d, 7 d, and 14 d after radiation, the learning and memory function of rats was evaluated. In this test, rats were placed at water level and then released into the water (temperature 19–23 ℃) at a specific location from four semirandom start positions as described in previous literature (Vorhees and Williams 2006). At the moment that the rats were released, the tracking program was started. If rats arrived at the platform that was submerged 1.5 cm below the water surface and remained in that location, the program was stopped. The time limit was 1 min per trial. Each session consisted of four trials. The average escape latency (AEL) of rats was recorded after a session.

### EEG Recording

EEG was used to record the brain electrophysiological activity of rats at 1 d, 7 d, 14 d after exposure. Briefly, rats were under light anaesthesia conditions, and then a four-electrode configuration was placed on the surface of the scalp. The EEG signals of rats were obtained by a BIOPACMP-150 system (BIOPAC, USA). The power spectral analyses of EEG signals were performed using spontaneous EEG segments.

### Sample collection

At 1 d after exposure, rats were anaesthetized using intraperitoneal injections of 1% pentobarbital sodium (50 mg/kg) and then euthanized. Brains were quickly removed. One-half of the brain was fixed in 4% paraformaldehyde for haematoxylin–eosin (HE) staining. The hippocampus (volume of 1 mm^3^) in another half of the brain was harvested for ultrastructure observation by transmission electron microscopy (TEM). The remaining part of the hippocampus was immediately frozen at -80 °C for the molecular experiments.

### HE staining

The microstructure damage in the rat hippocampus was assessed by quantitative analysis of HE staining results (the count of deeply stained neuron nuclei). Briefly, brains were dehydrated and embedded in paraffin at 1 d after exposure. Subsequently, the paraffin-embedded brain tissue was cut into 5 μm thick coronal sections. Every section was placed on a slide and stained with HE (ZSGB-BIO, China). The observation of the hippocampal microstructure was performed under light microscopy (Leica, Germany). The count of deeply stained neuronal nuclei in the hippocampus was calculated by the ImageJ version 1.48v program.

### TEM analysis

The cubes of rat hippocampus were fixed in 3% glutaraldehyde for 2 h and subsequently treated with 1% osmium tetroxide at 4 °C. After washing three times with 0.075 M PBS + 0.19 M sucrose, the cubes were dehydrated by graded ethyl alcohols at 4 °C and then embedded in EPON618. The sections were stained with uranyl acetate and lead citrate for 10 min. The hippocampal ultrastructure was observed under a TEM (HT7800, Hitachi, Japan).

### Iron level assessment

The ferrous ion (Fe^2+^) levels in the hippocampus were measured using iron assay kits (Sigma‒Aldrich, USA) following the manufacturer’s protocol. In brief, the Fe^2+^ of the hippocampus was released by an acidic buffer and then reacted with a chromogen to generate a colorimetric product. The Fe^2+^ levels were quantified using the absorbance at 593 nm on a SpectraMax 190 plate reader (Molecular Devices, San Jose, CA, USA).

### Lipid peroxidation assessment

The lipid peroxidation malonaldehyde (MDA) levels in the hippocampus were measured using commercial assay kits (Sigma‒Aldrich, St. Louis, MO, USA) following the manufacturer’s protocol. Briefly, the level of lipid peroxidation was estimated by the reaction of MDA with thiobarbituric acid (TBA) to generate a colorimetric product that was proportional to the amount of MDA. The absorbance at 532 nm was used to detect lipid peroxidation levels on a SpectraMax 190 plate reader (Molecular Devices, San Jose, CA, USA).

### Quantitative real-time PCR analysis

Total RNA was extracted from the hippocampus with TRIzol. First-strand cDNA was synthesized by Maxima Reverse Transcriptase (Thermo Fisher Scientific, Carlsbad, CA, USA). Quantitative real-time polymerase chain reaction (qRT‒PCR) was performed with SG Fast qPCR Master Mix (2x, Sangon Biotech, Shanghai, China) and the LightCycler480 II Real-Time PCR System (Roche, Rotkreuz, Switzerland). GAPDH was used as an internal reference control. The primer sequences used were as follows: PTGS2 5’-GCTTCTCCCTGAAACCTTACAC-3’ and 5’-TGGTCTCCCCAAAGATAGCA-3’; GAPDH 5’-CAAGTTCAACGGCACAGTCAA-3’ and 5’- CGCCAGTAGACTCCACGACA-3’.

### Western blotting

The rat hippocampus was lysed with RIPA buffer containing 1% protease inhibitor cocktail to extract total protein using a tissue homogenizer. After extraction, the concentration of protein was determined using the BCA method by commercial assay kits (Thermo Fisher Scientific, Carlsbad, CA, USA) at an absorbance of 562 nm. The total protein was added to a Jess separation kit (12–230 kDa, Protein Simple) following the manufacturer’s method. The expression of GPX4 protein was detected and analysed on the JESS System (Protein Simple).

### Statistical analysis

Quantitative data are presented as the mean ± SEM. SPSS 19.0 software (IBM, Armonk, NU, USA) was used for statistical analyses. The changes in rat rectum temperature before and immediately after exposure were analysed by paired Student’s t test. The difference among the different groups was analysed by one-way analysis of variance (ANOVA), followed by LSD’s post hoc test. Three-way repeated-measures ANOVA was used to investigate the interaction effect between MW, EMP and time on learning and memory function and brain oscillations. Two-way ANOVA was used to investigate the interaction effect between MW and EMP on the count of damaged hippocampal neurons, Fe^2+^ level, lipid peroxidation level, PTGS2 mRNA level, and GPX4 protein expression in the rat hippocampus. The Fe^2+^ level, lipid peroxidation level, PTGS2 mRNA level, and GPX4 protein expression were normalized to those of the Sham group. *p* < 0.05 was considered statistically significant.

## Results

### Rectum temperature in rats increased after MW exposure and EMP + MW combined exposure

**Fig. 2:**
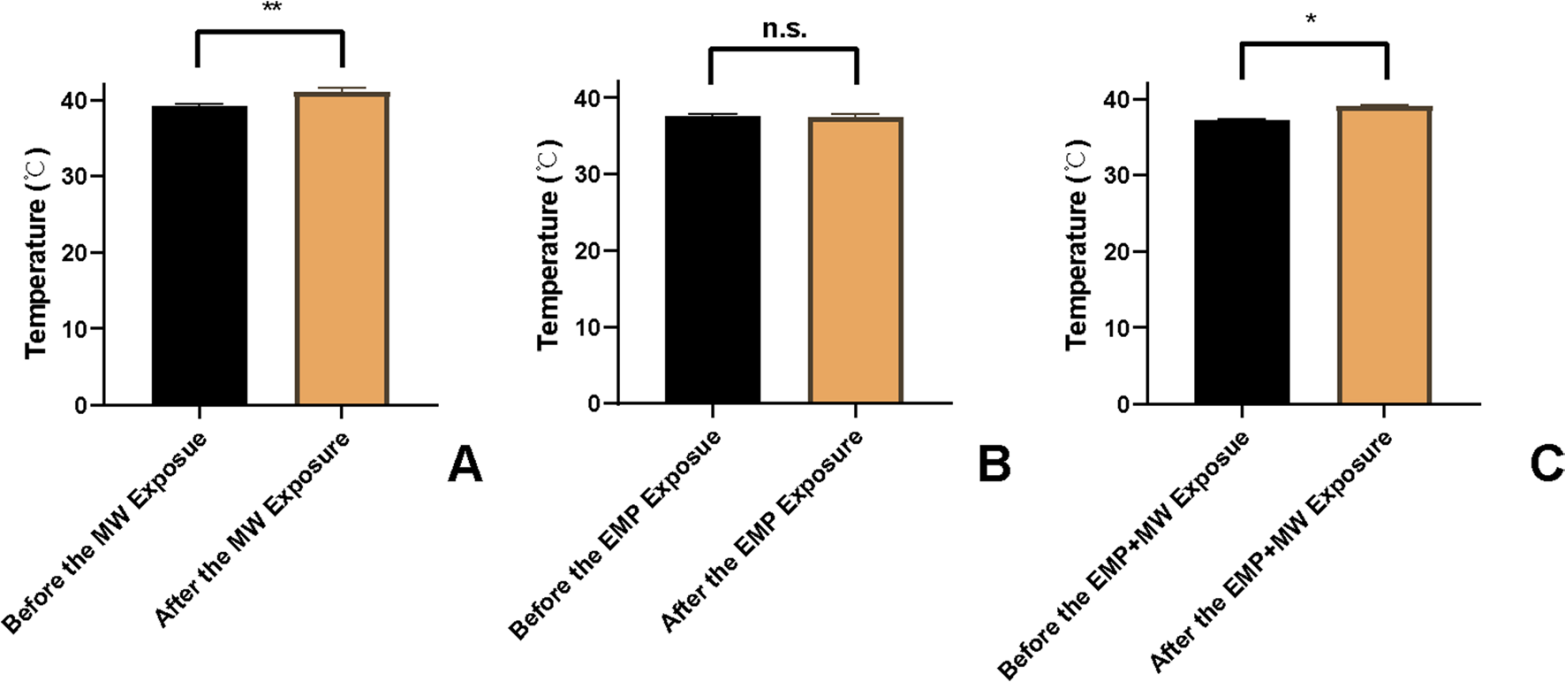
MW and EMP combined exposure increased the rectum temperature in rats (*n* = 3). The temperature in the rat rectum was measured before and immediately after (**A**) MW exposure, (**B**) EMP exposure, and (**C**) EMP + MW exposure. n.s., not significant; ^*^ *p* < 0.05; ^**^ *p* < 0.01

The rectum temperatures of rats were examined before and immediately after exposure using a fibre optic thermometer. As shown in Fig. 2 A and C, the temperature in the rat rectum was significantly increased after either MW or EMP + MW exposure (*p* < 0.01 or *p* < 0.05, respectively). No significant changes in rat rectum temperatures were found before and immediately after EMP exposure (*p* > 0.05, Fig. 2 B).

### Spatial learning and memory of rats declined after single and combined exposure to MW and EMP radiation

**Fig. 3:**
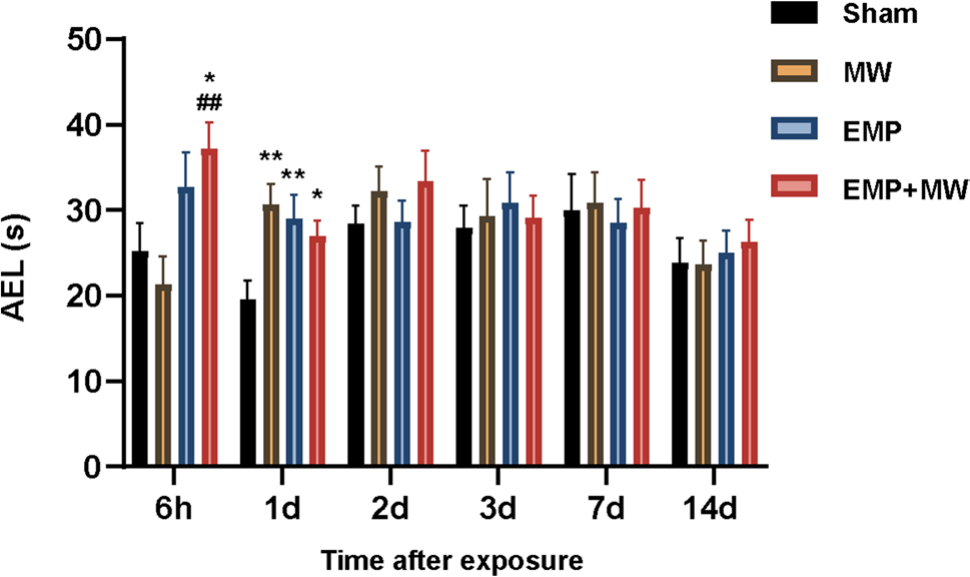
MW and EMP combined exposure caused the impairment of learning and memory function in rats. The MWM test was conducted at 6 h, 1–3 d, 7 d and 14 d following MW, EMP, and EMP + MW exposure (*n* = 10). ^*^ *p* < 0.05, ^**^ *p* < 0.01 vs. the Sham group; ^##^ *p* < 0.01 vs. the MW group

To assess the effects of the single and combined exposures on learning and memory, the AELs of the rats in each radiation group were compared with those in the Sham group. As shown in Fig. 3, compared with the Sham group, (1) the AELs in the EMP + MW group were significantly increased at 6 h (*p* < 0.05), and (2) the AELs in the MW, EMP, and EMP + MW groups were significantly increased at 1 d (*p* < 0.01, *p* < 0.01 or *p* < 0.05, respectively).

Moreover, to explore the effects between the single and combined exposures on learning and memory, the AELs in the MW + EMP group were compared with those in the MW group and EMP group. The AELs in the EMP + MW group showed a significant increase compared with those in the MW group at 6 h (*p* < 0.01). No significant difference in AELs was found between the EMP + MW group and the MW group (*p* > 0.05) or between the EMP + MW group and EMP group (*p* > 0.05) at each timepoint.

Furthermore, a three-way repeated-measures ANOVA was conducted to assess the interaction effect of MW × EMP × time on AELs after exposure. There was no statistically significant interaction effect among MW, EMP and time on AELs (*p* > 0.05, Fig. 3). No significant simple two-way interaction between EMP and MW, EMP and time, or MW and time was observed (*p* > 0.05, *p* > 0.05 or *p* > 0.05, Fig. 3, respectively).

### Brain oscillations in rats altered after single and combined exposure to MW and EMP radiation

**Fig. 4:**
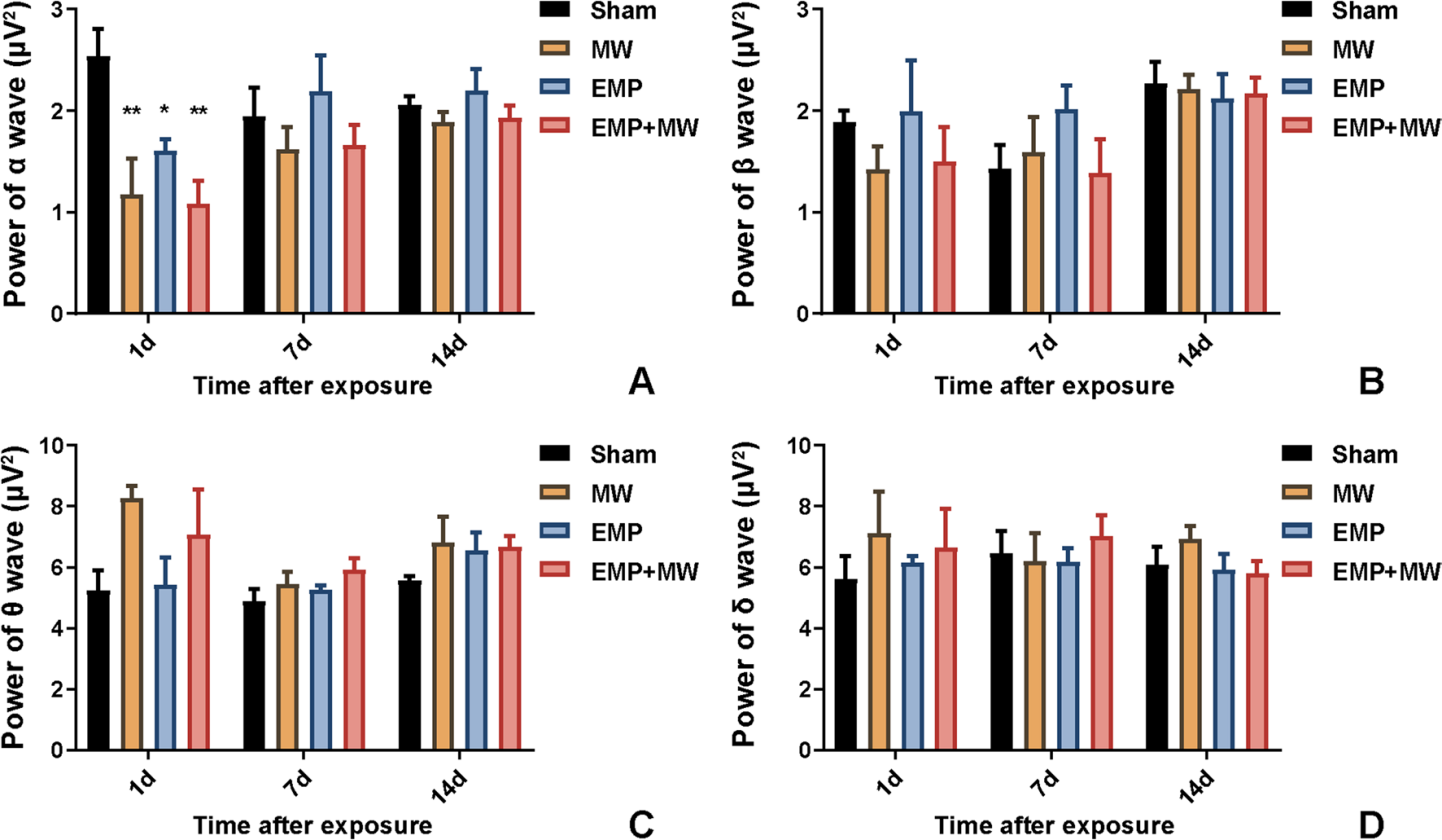
Combined MW and EMP exposure caused alterations in brain oscillations in rats (*n* = 3). (**A**) The powers of the α wave, (**B**) the powers of the β wave, (**C**) the powers of the θ wave, and (D) the powers of the δ wave were measured in rats after MW and EMP exposure. ^*^ *p* < 0.05, ^**^ *p* < 0.01 vs. the Sham group

To assess the changes in brain oscillations of rats that were associated with learning and memory ability, the powers of α, β, θ, and δ waves were recorded at 1 d, 7 d, and 14 d after exposure. In comparison with the Sham group, MW, EMP and EMP + MW exposure significantly decreased the power of the α wave at 1 d (*p* < 0.01, p < 0.05 or *p* < 0.01, respectively, Fig. 4 A).

Compared with the MW group, no significant changes in brain oscillations were found after EMP + MW exposure (*p* > 0.05, Fig. 4 A-D). Compared with the EMP group, the EMP + MW group did not show significant differences in brain oscillations after exposure (*p* > 0.05, Fig. 4 A-D).

Three-way repeated-measures ANOVA was conducted to assess the interaction effect of MW × EMP × time on the powers of α, β, θ, and δ waves after exposure. There was no significant interaction effect between MW, EMP and time on the powers of α, β, θ, and δ waves after exposure (*p* > 0.05, Fig. 4 A-D). No significant simple two-way interaction between EMP and MW, EMP and time, or MW and time was observed (*p* > 0.05, *p* > 0.05 or *p* > 0.05, Fig. 4 A-D, respectively).

### Pathological injuries of the hippocampus in rats caused by single and combined exposure to MW and EMP radiation

**Fig. 5:**
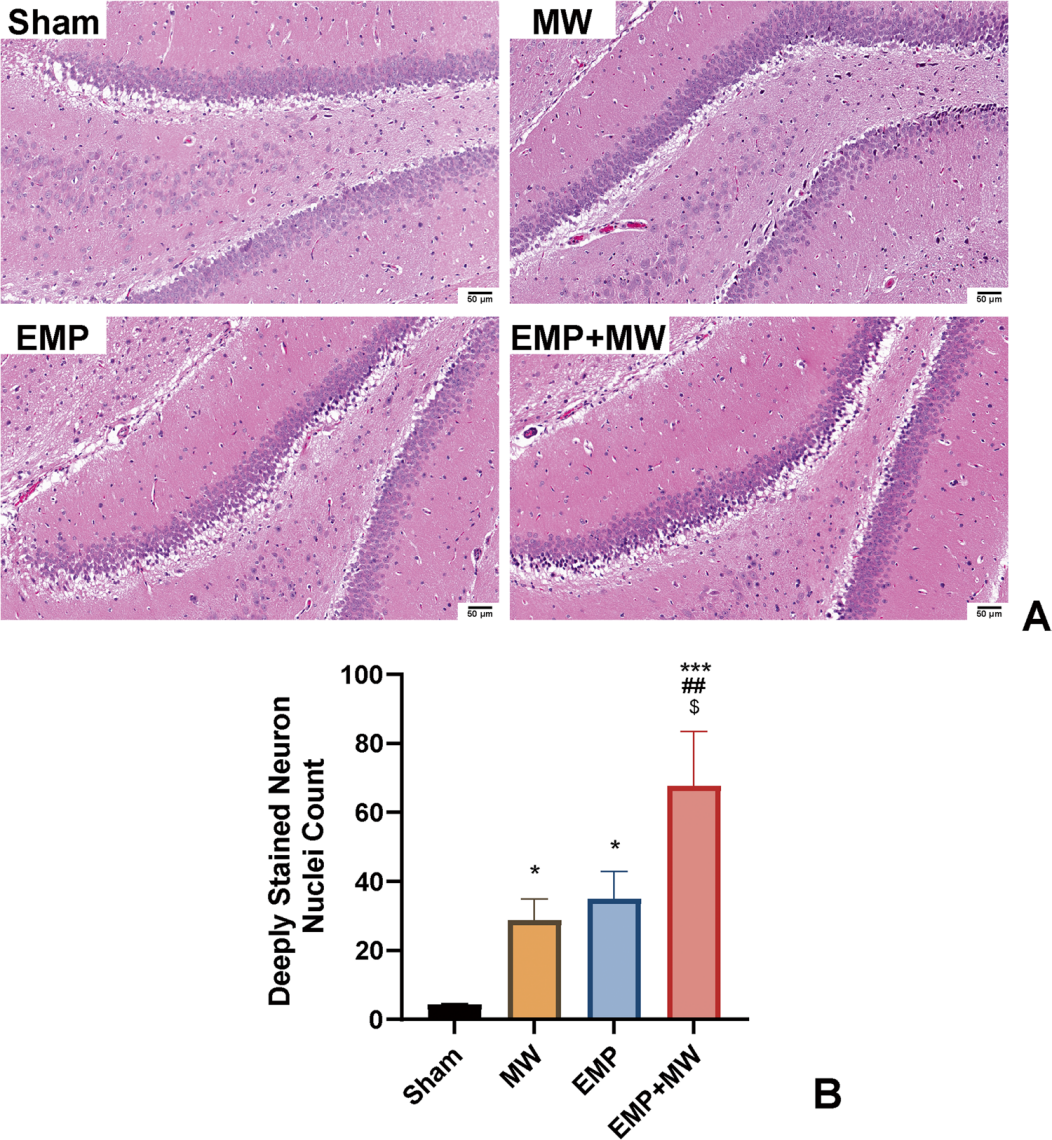
Combined MW and EMP exposure caused microstructural injuries in the rat hippocampus. (**A**) Representative images from brains stained with HE in the Sham, MW, EMP, and EMP + MW groups at 1 d after exposure (scale bar = 50 μm). (**B**) The counts of deeply stained neuronal nuclei were estimated in the DG area of the hippocampus after MW, EMP and EMP + MW exposure (Sham: n = 4, MW: *n* = 4, EMP: *n* = 4, EMP + MW: *n* = 3). ^*^ *p* < 0.05, ^***^ *p* < 0.001 vs. the Sham group; ^##^ *p* < 0.01 vs. the MW group; ^$^ *p* < 0.05 vs. the EMP group

To assess the effect of MW and EMP-induced microstructural injuries of the hippocampus, we performed HE staining in the rat hippocampus at 1 d after exposure. Regarding the results of HE staining, compared with the Sham group, all exposure groups showed obvious damage in the hippocampus after exposure (Fig. 5). The microstructural injury changes in the hippocampus included karyopyknosis and irregular arrangement of neurons around the dentate gyrus (DG) area of the hippocampus. The count of deeply stained neuron nuclei was used to quantitatively analyse hippocampal microstructural damage. Compared with the MW or EMP group, increased counts of deeply stained neuron nuclei were observed in the EMP + MW group (*p* < 0.01 or *p* < 0.05, Fig. 5, respectively). Two-way ANOVA was implemented to detect the interaction effect of MW × EMP on the microstructural changes following exposure. There was no significant interaction effect between MW and EMP after exposure (*p* > 0.05).

**Fig. 6:**
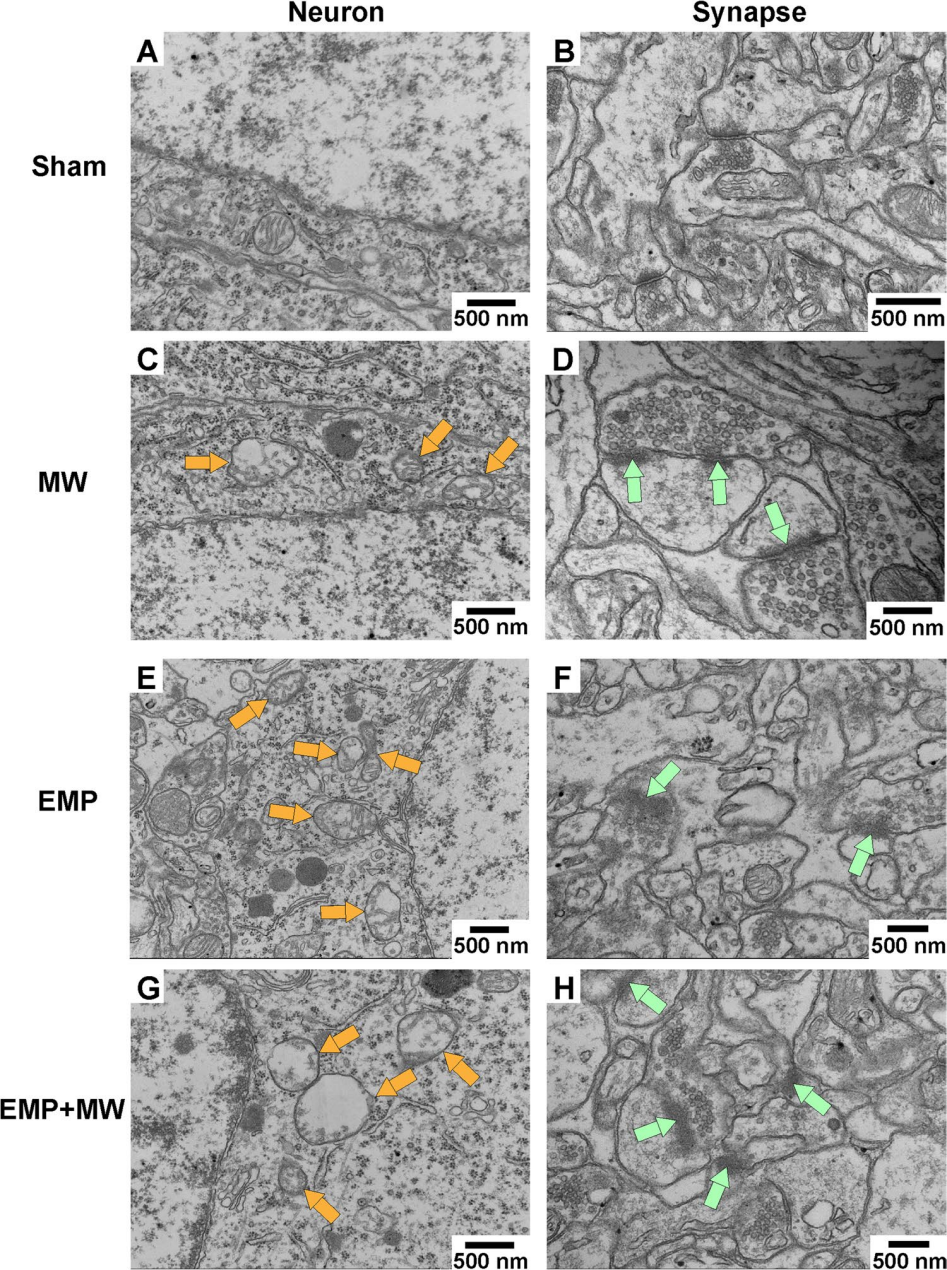
MW and EMP combined exposure caused ultrastructural injuries in the rat hippocampus (*n* = 3 for each group, scale bar = 500 nm). (**A**, **B**) Representative TEM images from the rat hippocampus showing the normal ultrastructure of neurons (left column) and synapses (right column) in the Sham group. (**C**-**H**) Representative TEM images showing the damaged ultrastructure of hippocampal neurons (left column) and synapses (right column) in the MW, EMP, and EMP + MW groups. Orange arrows indicated swollen mitochondria with reduced numbers of cristae. Green arrows indicated blurred synaptic clefts

The ultrastructural changes in the rat hippocampus were observed by TEM at 1 d after exposure. As shown in Fig. 6 C-H, compared with the Sham group, obvious ultrastructural damage to the hippocampus was found in the MW, EMP, and EMP + MW groups. The injury changes showed abnormal hippocampal neurons, including swollen mitochondria, reduced numbers of mitochondrial cristae, and blurred synaptic clefts. The most serious damage was found in the EMP + MW group.

### Changes in ferroptosis hallmarks in the rat hippocampus induced by single and combined exposure to MW and EMP radiation

**Fig. 7:**
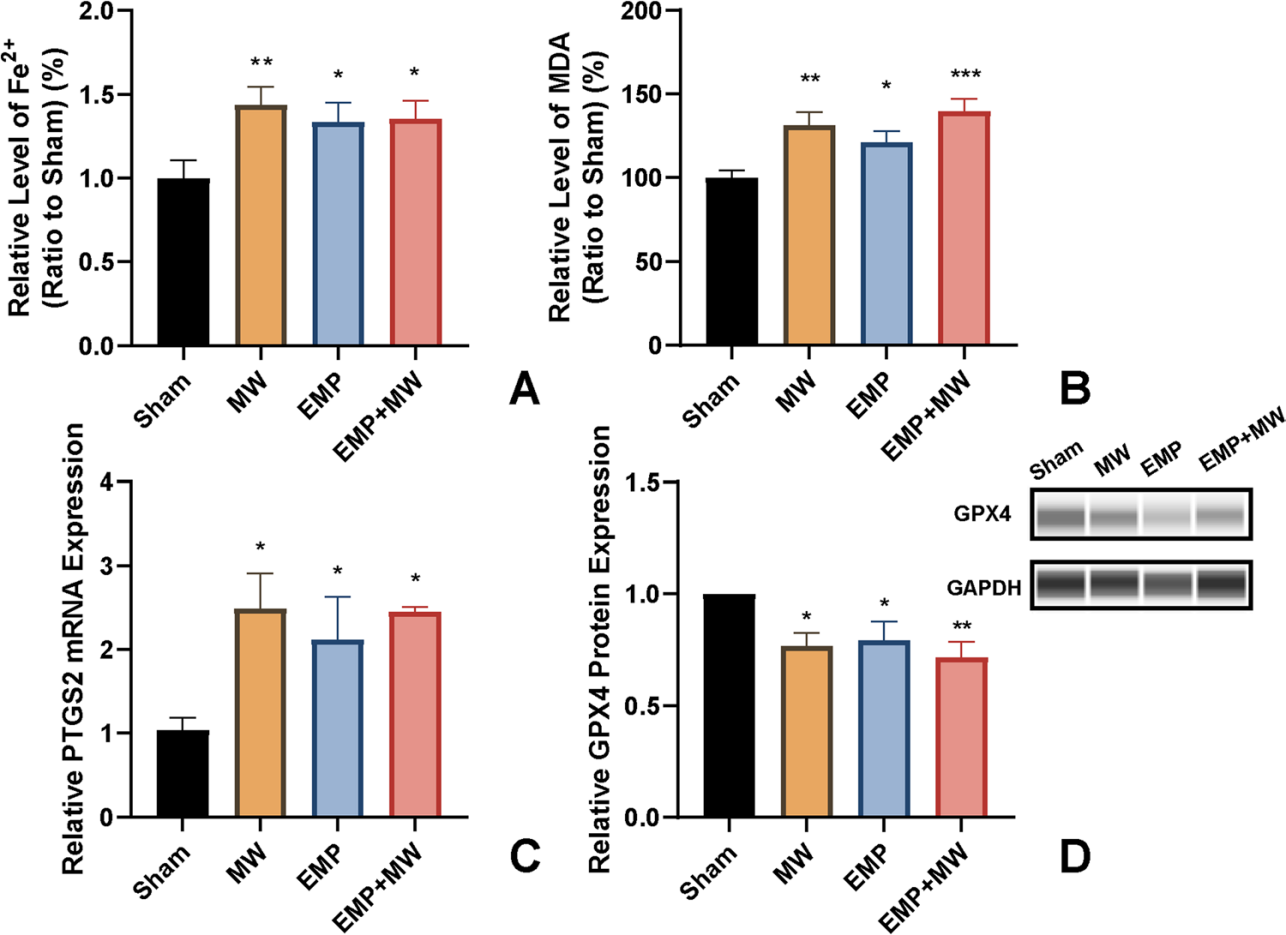
Combined MW and EMP exposure induced changes in ferroptosis hallmarks in the rat hippocampus. The levels of (**A**) Fe^2+^ (Sham: *n* = 9; MW: *n* = 9; EMP: *n* = 8; EMP + MW: n = 9), (**B**) MDA (Sham: *n* = 8; MW: *n* = 10; EMP: *n* = 10; EMP + MW: *n* = 10), (**C**) PTGS2 mRNA (*n* = 4 for each group), and (**D**) GPX4 protein (*n* = 5 for each group) were detected following MW, EMP and EMP + MW exposure. ^*^ *p* < 0.05, ^**^ *p* < 0.01, ^***^ *p* < 0.001 vs. the Sham group

To estimate the association between ferroptosis and hippocampal injury after exposure, the putative biomarkers of ferroptosis, including Fe^2+^, MDA, PTGS2 mRNA, and GPX4 protein, were detected in the hippocampus. In comparison with the Sham group, (1) MW exposure caused significantly increased levels of Fe^2+^ (*p* < 0.01, Fig. 7 A) and MDA (*p* < 0.01, Fig. 7 B) and elevated expression of PTGS2 mRNA (*p* < 0.05, Fig. 7 C) as well as decreased expression of GPX4 protein (*p* < 0.05, Fig. 7 D); (2) EMP exposure also significantly increased levels of Fe^2+^ (*p* < 0.05, Fig. 7 A) and MDA (*p* < 0.05, Fig. 7 B) and elevated expression of PTGS2 mRNA (*p* < 0.05, Fig. 7 C) as well as decreased GPX4 protein expression (*p* < 0.05, Fig. 7 D); and (3) MW and EMP combined exposure caused significantly increased levels of Fe^2+^ (*p* < 0.05, Fig. 7 A), MDA (*p* < 0.001, Fig. 7 B) and mRNA expression of PTGS2 (*p* < 0.05, Fig. 7 C) as well as decreased GPX4 protein expression (*p* < 0.01, Fig. 7D).

Compared with the MW group, no significant changes in Fe^2+^ (*p* > 0.05), MDA (*p* > 0.05), PTGS2 mRNA (*p* > 0.05) or GPX4 protein (*p* > 0.05) were found after EMP + MW exposure (Fig. 7). Compared with the EMP group, there were also no significant changes in Fe^2+^ (*p* > 0.05), MDA (*p* > 0.05), PTGS2 mRNA (*p* > 0.05) and GPX4 protein (*p* > 0.05) after EMP + MW exposure (Fig. 7).

Two-way ANOVA was used to detect the interaction effect of MW × EMP on the levels of Fe^2+^, MDA, PTGS2 mRNA, and GPX4 protein after exposure. There was no significant interaction effect between MW and EMP on the levels of Fe^2+^, MDA, PTGS2 mRNA, and GPX4 protein after exposure (*p* > 0.05, *p* > 0.05, *p* > 0.05 or *p* > 0.05, Fig. 7, respectively).

## Discussion

The adverse effects of electromagnetic radiation have aroused public concern due to the ubiquitous use of electronic products in modern society. Although many efforts have been made to investigate the bioeffects induced by a single exposure to electromagnetic radiation (Lai et al. 2021), it is more important to explore the effects of combined exposure to different types of electromagnetic radiation, which represents a more realistic environment. In this study, for the first time, we demonstrated that MW and EMP combined radiation could cause learning and memory impairment and hippocampal damage in rats. Moreover, no interaction effects between MW and EMP radiation were observed. Furthermore, we also found that ferroptosis in the hippocampus was potentially a common underlying mechanism of learning and memory impairment induced by both single or combined MW and EMP exposure.

Previous studies demonstrated that either MW or EMP single exposure at certain doses could cause learning and memory loss (Deshmukh et al. 2015; Zhu et al. 2021; Zhi et al. 2018; Shahin et al. 2018; Lai et al. 2022). Zhu RQ et al. (Zhu et al. 2021) found that 10 mW/cm^2^ 1.5 GHz MW exposure led to learning and memory impairments and hippocampal structure damage in rats. Our previous works reported that in vivo exposure of rats to EMP radiation at 400 ± 25 kV/m (total 400 pulses) impaired learning and memory abilities (Lai et al. 2022). In our study, we found that either 1.5 GHz MW radiation or 400 kV/m EMP single exposure could induce learning and memory decline, which was consistent with these previous studies. However, the effects of combined exposure to different types of electromagnetic radiation have never been investigated. MW and EMP, well-known electromagnetic waves, possess different physical properties. Our current study demonstrated that the combination of MW and EMP exposure could also induce learning and memory impairment in rats.

Brain oscillations have been proven to play a functional role in cognition (Giustiniani et al. 2022). Alterations in brain oscillations are linked to the progression of cognitive decline in some neurological diseases, such as Alzheimer’s disease (AD), frontotemporal degeneration (FTD), and vascular dementia (VaD) (Giustiniani et al. 2022). It has been reported that MW radiation could impair the learning and memory of rats along with the decreased power of α band oscillations and the increased θ band activities (Wang et al. 2017; Hao et al. 2018; Li et al. 2015). In our study, similar results were observed for 1.5 GHz MW radiation. Moreover, the effects of EMP radiation remain controversial. Li J et al. (Li et al. 2003) found that 200 kV/m EMP radiation (repetition rate of 0.5 Hz for 200 pulses) caused changes in the electrophysiological activity of the rat brain. In contrast, Mattsson JL et al. (Mattsson and Oliva 1976) reported that 266 kV/m EMP radiation (repetition rate of 5 pps Hz for 18,700 pulses) had no effects in rhesus monkeys. Our results showed that the power of α band brain oscillations decreased concurrently with the decline in learning and memory. The conflicts between our results and the negative observation in the previous monkey study might rely on the differences in the physical parameters of EMP and the species of animal model. Furthermore, combined MW radiation with multiple frequencies was reported to be harmful to the brain electrophysiological activity of rats (Tan et al. 2017; Zhu et al. 2021). In this study, we observed decreased power of α band brain oscillations in rats along with learning and memory deficits following combined exposure to MW and EMP radiation. All of these results implied that either single or combined MW and EMP radiation could induce learning and memory impairment via alteration of brain electrophysiological activity.

Learning and memory abilities are known to be closely associated with the status of hippocampal neurons (Bettio et al. 2017; Eichenbaum 2000). Single exposure to either MW or EMP radiation was found to be responsible for structural damage to hippocampal neurons in rats (Li et al. 2019; Zhu et al. 2021; Saikhedkar et al. 2014). Neurodegeneration in the hippocampus is one of the reasons for the learning and memory decline induced by MW and EMP single radiation (Li et al. 2019; Zhu et al. 2021; Saikhedkar et al. 2014). In this study, deeply stained nuclei, damage to mitochondria, and blurred synaptic clefts were observed in the rat hippocampus after either single or combined exposure to MW and EMP radiation. Considering the functions of the hippocampus, these results suggested that damage to hippocampal neurons accounted for the learning and memory decline induced by either single or combined exposure to MW and EMP radiation. Moreover, the damage to hippocampal neurons induced by the combined radiation was more severe than that induced by single exposures to either MW or EMP radiation.

Ferroptosis is a recently identified form of regulated cell death (RCD) caused by excessive oxidative stress. Ferroptosis is responsible for various neurodegenerative diseases (Zou and Schreiber 2020; Zhang et al. 2020; Alim et al. 2019). MW exposure alone can activate oxidative stress in neurons (Shahin et al.^2019^; Sharma et al. 2017a, 2017b; Marjanovic Cermak et al. 2017). Although some alterations in oxidative stress factors, such as the reduced level of glutathione peroxidase (GPX) and the increased level of the lipid peroxidation product MDA, have been found following single MW exposure (Shahin et al. 2019; Sharma et al. 2017a, 2017b; Marjanovic Cermak et al. 2017), the involvement of ferroptosis in the pathophysiological process of single MW exposure has never been examined. Single EMP exposure was demonstrated to induce hippocampal neuronal ferroptosis in our previous studies (Lai et al. 2022). In this work, we found that either single or combined exposure to MW and EMP radiation could cause changes in ferroptosis hallmarks, including decreased GPX4 expression, increased levels of PTGS2 mRNA, and accumulation of Fe^2+^ and MDA in the rat hippocampus. Moreover, significant abnormal mitochondrial morphology, including swollen mitochondria with reduced cristae, was also observed in rat hippocampal neurons after single or combined exposure. These results suggested that ferroptosis in hippocampal neurons might be a common underlying mechanism of learning and memory impairment induced by both single and combined MW and EMP exposure.

Although some efforts have been made to investigate the biological effects of combined MW radiation (Shirai et al. 2017; Lee et al. 2016; Kang et al. 2014), only a few of them have been concerned with the interaction effects between single MW radiation (Tan et al. 2017; Zhu et al. 2021). For instance, Tan SZ et al. (Tan et al. 2017) reported a possible aggravated interaction effect between 2.856 GHz and 1.5 GHz MW on the content of Nissl substances and AchE expression in the rat hippocampus. Zhu RQ et al. (Zhu et al. 2021) found no significant interaction effect of 1.5 GHz and 4.3 GHz MW radiation on learning and memory function in rats. However, the interaction effects of MW and EMP radiation have never been examined. In this paper, although the pathological examination demonstrated that the damage to hippocampal neurons induced by the combined radiation was more severe than the single exposures, no significant interaction effects between MW and EMP were found. These results suggested that the heavier damage to hippocampal neurons induced by the combined exposure might rely on the cumulative effects of MW and EMP radiation rather than their synergistic effects.

## Conclusion

In conclusion, either single or combined exposure to MW and EMP radiation could impair learning and memory and damage hippocampal neurons in rats. Moreover, the injury effects on the hippocampus caused by combined exposure were more severe than those caused by either MW or EMP exposure alone. This might rely on the cumulative effects of the single exposures rather than their synergistic effects. Interestingly, ferroptosis in the hippocampus could be a common underlying mechanism of learning and memory impairment induced by both single and combined MW and EMP exposure.

## Data Availability

The data and materials from this work could be available on reasonable request.
